# Modes of Death in Patients with Cardiogenic Shock in the Cardiac Intensive Care Unit: A Report from the Critical Care Cardiology Trials Network

**DOI:** 10.1016/j.cardfail.2024.01.012

**Published:** 2024-02-21

**Authors:** DAVID D. BERG, SACHIT SINGAL, MICHAEL PALAZZOLO, VIVIAN M. BAIRD-ZARS, FADEL BOFARRAG, ERIN A. BOHULA, SUNIT-PREET CHAUDHRY, MARK W. DODSON, DUSTIN HILLERSON, PATRICK R. LAWLER, SHUANGBO LIU, CONNOR G. O’BRIEN, BARBARA A. PISANI, LEKHA RACHARLA, ROBERT O. ROSWELL, KEVIN S. SHAH, MICHAEL A. SOLOMON, LAKSHMI SRIDHARAN, ANDREA D. THOMPSON, SEAN VAN DIEPEN, JASON N. KATZ, DAVID A. MORROW

**Affiliations:** 1 Levine Cardiac Intensive Care Unit, Cardiovascular Division, Department of Medicine, Brigham and Women’s Hospital, Harvard Medical School, Boston, Massachusetts; 2 Department of Medicine, St Vincent Heart Center, Indianapolis, Indiana; 3 Department of Pulmonary and Critical Care Medicine, Intermountain Medical Center, Murray, Utah; 4 Department of Medicine, Division of Cardiovascular Medicine, University of Wisconsin School of Medicine and Public Health, Madison, Wisconsin; 5 McGill University Health Centre, Montreal, Quebec, Canada; 6 Section of Cardiology, Department of Internal Medicine, Max Rady College of Medicine, Rady Faculty of Health Sciences, University of Manitoba, Winnipeg, Manitoba, Canada; 7 Division of Cardiology, Department of Medicine, University of California San Francisco, San Francisco, California; 8 Section of Cardiovascular Medicine, Department of Internal Medicine, Wake Forest Baptist Medical Center, Winston-Salem, North Carolina; 9 Lehigh Valley Heart Institute, Allentown, Pennsylvania; 10 Northwell, Department of Cardiology, Zucker School of Medicine at Hofstra/Northwell. New Hyde Park, NY; 11 Division of Cardiology, Department of Medicine, University of Utah, Salt Lake City, Utah; 12 Critical Care Medicine Department, National Institutes of Health Clinical Center and Cardiovascular Branch, National Heart, Lung, Blood Institute of the National Institutes of Health, Bethesda, Maryland; 13 Division of Cardiovascular Medicine, Department of Medicine, Emory University School of Medicine, Atlanta, Georgia; 14 Division of Cardiovascular Medicine, Department of Medicine, University of Michigan, Ann Arbor, Michigan; 15 Department of Critical Care Medicine and Division of Cardiology, Department of Medicine, University of Alberta, Edmonton, Alberta, Canada; 16 NYU Grossman School of Medicine & Bellevue Hospital Center, New York, New York

**Keywords:** Cardiogenic shock, death, cardiac arrest, mechanical circulatory support

## Abstract

**Background:**

There are limited data on how patients with cardiogenic shock (CS) die.

**Methods:**

The Critical Care Cardiology Trials Network is a research network of cardiac intensive care units coordinated by the Thrombolysis In Myocardial Infarction (TIMI) Study Group (Boston, MA). Using standardized definitions, site investigators classified direct modes of in-hospital death for CS admissions (October 2021 to September 2022). Mutually exclusive categories included 4 modes of cardiovascular death and 4 modes of noncardiovascular death. Subgroups defined by CS type, preceding cardiac arrest (CA), use of temporary mechanical circulatory support (tMCS), and transition to comfort measures were evaluated.

**Results:**

Among 1068 CS cases, 337 (31.6%) died during the index hospitalization. Overall, the mode of death was cardiovascular in 82.2%. Persistent CS was the dominant specific mode of death (66.5%), followed by arrhythmia (12.8%), anoxic brain injury (6.2%), and respiratory failure (4.5%). Patients with preceding CA were more likely to die from anoxic brain injury (17.1% vs 0.9%; *P* < .001) or arrhythmia (21.6% vs 8.4%; *P* < .001). Patients managed with tMCS were more likely to die from persistent shock (*P* < .01), both cardiogenic (73.5% vs 62.0%) and noncardiogenic (6.1% vs 2.9%).

**Conclusions:**

Most deaths in CS are related to direct cardiovascular causes, particularly persistent CS. However, there is important heterogeneity across subgroups defined by preceding CA and the use of tMCS.

Cardiogenic shock (CS) is a life-threatening syndrome characterized by impaired cardiac output, systemic hypoperfusion, and multi-organ dysfunction. Despite advances in CS management, estimated mortality from CS remains high (30%–50%).^1^ CS is clinically heterogeneous with distinct underlying etiologies (eg, acute myocardial infarction [AMI], decompensated heart failure [HF]), diverse hemodynamic profiles, and widely ranging severity. Although our understanding of CS epidemiology has expanded, there are limited data describing how patients with CS die, which, depending on potential for mitigation, may have implications for developing new therapeutic strategies, optimizing clinical trial designs, and improving CS outcomes. For example, anoxic encephalopathy and respiratory failure are less likely to be modified by cardiovascular therapies and, even among cardiovascular drivers of mortality, dominant hemodynamic vs electrical instability may require distinct treatment prioritization.^2^ In addition, understanding patterns of CS mortality may have implications for temporary mechanical circulatory support (tMCS) device selection as well as identification of those likely to derive benefit from destination cardiac replacement therapies (eg, durable ventricular assist devices).

Accordingly, we performed a nested prospective study within a multinational registry of cardiac critical illness to describe the primary modes of in-hospital death among patients admitted with CS to cardiac intensive care units (CICUs).

## Methods

### Study Population

The Critical Care Cardiology Trials Network (CCCTN) is a multinational research network of CICUs with oversight by the Thrombolysis In Myocardial Infarction (TIMI) Study Group (Boston, MA). Methods for the CCCTN Registry have been described.^3^ From 2021 to 2022, 32 centers contributed data on consecutive medical CICU admissions during annual 2-month collection periods. In addition, year-round capture of consecutive admissions was permitted.

The present analysis included patients with CS assessed by site investigators using standardized definitions.^1^ Patients with coronavirus disease 2019 were excluded to ensure that the results were not overly influenced by an intercurrent event that might be unique to a limited time period. CS etiology was subdivided into AMI-CS, HF-CS, and secondary (nonmyocardial) CS (eg, tamponade). Subgroups defined by CS type, cardiac arrest (CA) before CICU admission, use of tMCS, and transition to comfort measures only (CMO) were evaluated.

### Death Classification

For the annual collection cycle from October 2021 to September 2022, site investigators classified the dominant mode of in-hospital death for all patients with CS who died using standardized definitions (Table 1). These consensus definitions were developed by CCCTN leadership for prospective application in the registry. Rather than identifying the proximate cause of death, investigators were prospectively trained to identify the direct reason for death (i.e., mode of exit), which may have been different from the initial diagnosis or indication for CICU admission. Mutually exclusive categories included 4 modes of cardiovascular death (CS, arrhythmia, stroke, or other cardiovascular death) and 4 modes of noncardiovascular death (respiratory failure, anoxic brain injury, noncardiogenic shock, or other noncardiovascular death). Classification of shock type (ie, cardiogenic vs noncardiogenic) was based on previously reported criteria.^1^

## Results

Among 1068 CS cases, 337 (31.6%) died during the index hospitalization with a median time to death of 4.4 days (Q1–Q3, 1.2–10.9 days) from CICU admission. Clinical characteristics are summarized in Table 2. One-third of patients with CS (32.9%) who died had CA before CICU admission, 39.2% were managed with tMCS, and 67.1% were transitioned to CMO. In addition, 22.0% evolved to a secondary shock state, including 11.6% who evolved to mixed shock (i.e., vasodilatory CS) and 7.4% who evolved to pure distributive shock.

**Fig. 1.**
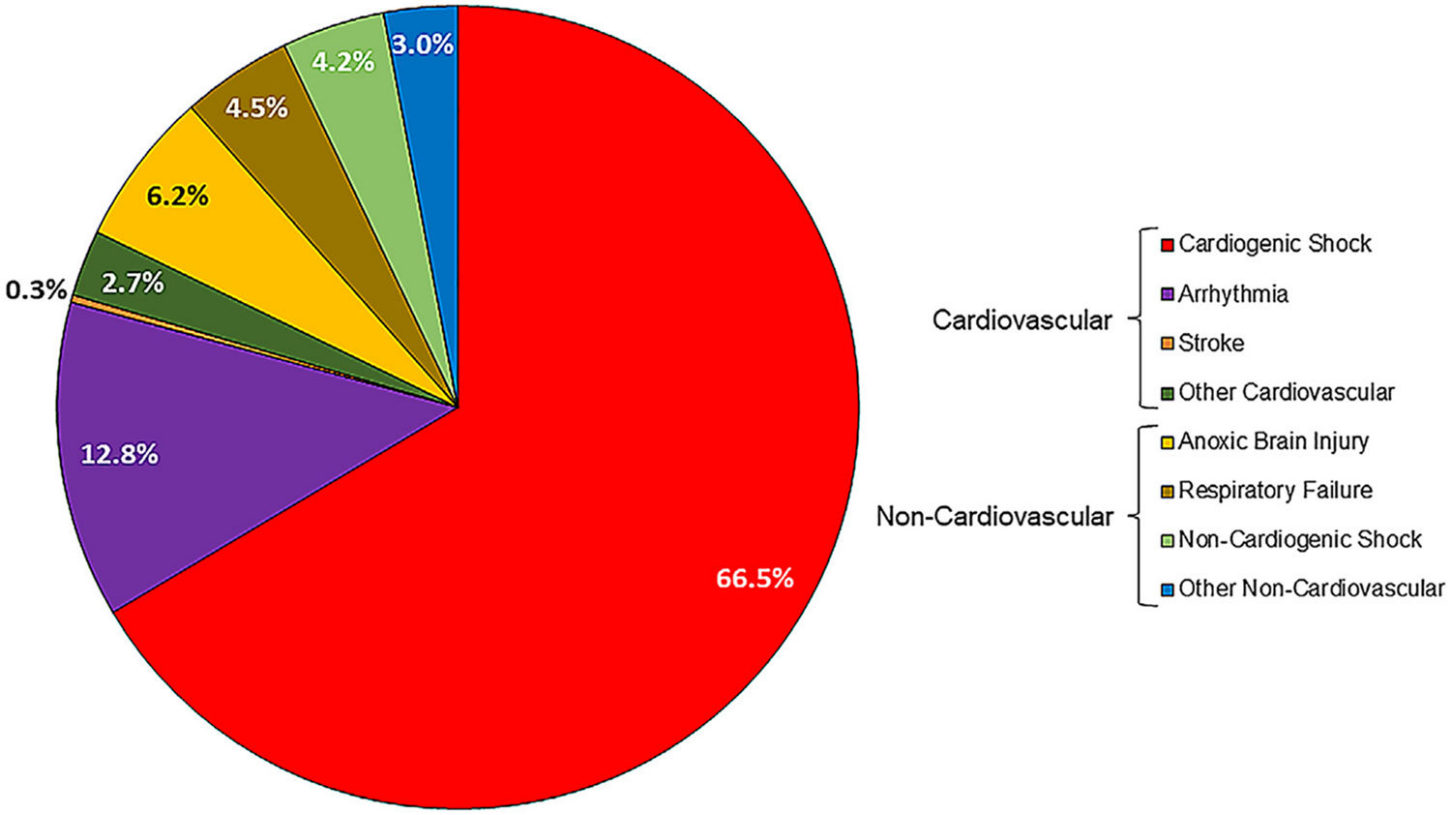
Modes of in-hospital death in patients with cardiogenic shock (*N* = 337).

Across all CS deaths, the primary mode of death was cardiovascular in 82.2% of cases. Persistent CS was the dominant mode of death (66.5% of deaths), followed by arrhythmia (12.8% of deaths). The leading modes of non-cardiovascular death were anoxic brain injury and respiratory failure, accounting for 6.2% and 4.5%, respectively, of deaths (Fig. 1).

**Fig. 2.**
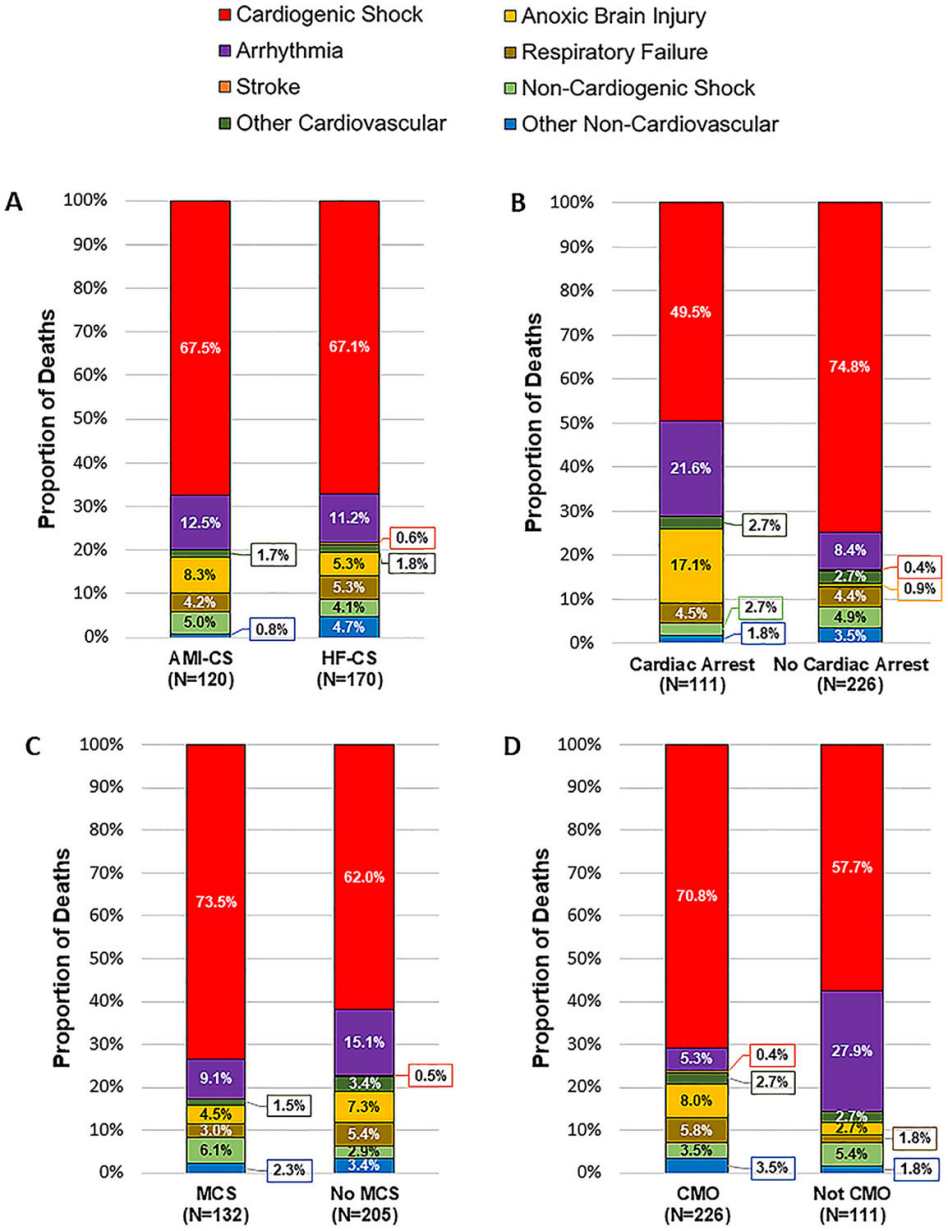
Modes of in-hospital death in patients with cardiogenic shock in key subgroups. Modes of in-hospital death are shown by: (A) cardiogenic shock subtype (secondary CS not shown owing to the small sample size [*n* = 47]), (B) cardiac arrest before CICU admission, (C) use of mechanical circulatory support during CICU admission, and (D) transition to CMO before death. AMI, acute myocardial infarction; CMO, comfort measures only; CS, cardiogenic shock; HF, heart failure; MCS, mechanical circulatory support.

There were no meaningful differences in modes of death between patients with AMI-CS and patients with HF-CS (Fig. 2). Compared with patients without preceding CA, those with CA before CICU admission were more likely to die from anoxic brain injury (17.1% vs 0.9%; *P* < .001) or arrhythmia (21.6% vs 8.4%; *P* < .001). Compared with patients managed without tMCS, those managed with tMCS were more likely to die from persistent shock (*P* < .01), including both cardiogenic (73.5% vs 62.0%) and noncardiogenic (6.1% vs 2.9%) shock. Last, compared with patients who were not transitioned to CMO before death, those who were transitioned to CMO were more likely to die from persistent CS (70.8% vs 57.7%; *P* = .016) and less likely to die from arrhythmia (5.3% vs 27.9%; *P* < .001) (Fig. 2).

## Discussion

In this analysis of modes of death in a broad CS population from a contemporary multinational registry, we found that most in-hospital deaths in patients with CS are cardiovascular and most are specifically related to persistent CS. These observations confirm the findings of prior analyses of patients with AMI-CS from both clinical trials and registries,^4,5^ and extend them to patients with HF-CS. In addition, among patients with CS after resuscitated CA, arrhythmic deaths and deaths owing to anoxic brain injury were found to be relatively more common, and among patients with CS managed with tMCS before death, persistent shock (both cardiogenic and noncardiogenic) was found to account for a greater majority of the deaths.

Defining modes of death is important for understanding the natural history of CS and for identifying opportunities to improve outcomes in this highly mortal syndrome. In the context of accumulating literature highlighting the extracardiac complications of CS and CS-related therapies, it is notable that persistent CS remains the dominant mode of death in CS. However, for several reasons, these results should not necessarily be interpreted as suggesting that more robust forms of hemodynamic support are critical to improving outcomes. First, two-thirds of CS deaths were preceded by a CMO transition, and, in that group, persistent CS was an especially common reason for death. In many of these cases, death was likely the result of an active transition in goals of care in the setting of persistent CS without a viable destination, rather than the inability to adequately support the patient with tMCS. Second, our data indicate the important contribution of noncardiogenic shock (eg, refractory vasoplegia) to CS mortality among those managed with tMCS, which is not an uncommon clinical evolution with tMCS. Finally, randomized trials of tMCS to date have uniformly failed to demonstrate improved survival with these devices.^6,7^ Nevertheless, our data emphasize the importance of reversing underlying drivers of CS and interrupting the vicious “cardiogenic shock spiral” that underpins mortality in CS,^8^ which should remain the focus of future interventional trials. At the same time, the fact that one-third of patients with CS died from a mode other than persistent CS underscores, in part, what makes intervening in CS so challenging.

Our data also highlight clinically important heterogeneity in how patients die with coincident CS and CA, namely, the greater prevalence of arrhythmic deaths and deaths owing to anoxic brain injury compared with those without preceding CA. These observations underscore the distinct insults and clinical sequelae associated with CA, which introduce competing risks that may affect the interpretation of clinical trials. Indeed, subgroup analyses of CS trials have suggested possible (albeit nonsignificant) heterogeneity in the treatment effects of certain therapies (eg, tMCS devices) according to CA status.^6,7^ To this end, there have been calls to stratify analyses by CA status in CS trials, and even to analyze these subgroups separately.^2^ Notably, the prevalence of preceding CA among patients with CS who died was lower than reported in recent trials.^7^ This finding is likely a function of trial entry criteria (eg, requirement for elevated serum lactate), which can lead to biased sampling of patients with CS enrolled in clinical trials. Our observations, thus, provide a more generalizable view of CS mortality and emphasize the importance of enrolling a sufficient number of patients without preceding CA in CS trials.

Several limitations of our study should be acknowledged. First, death classification was not adjudicated formally by a central clinical events committee. Nevertheless, site investigators were trained prospectively on standardized criteria for classifying modes of death. Second, accurately classifying mode of death in CS is complicated, not only because it is often multifactorial in patients with multiorgan system dysfunction, but because assigning a single mode of death may not adequately account for the clinical nuances involved in a patient’s death. For example, a patient with ongoing or worsening hemodynamic instability and multiorgan system dysfunction for whom care is withdrawn may be classified as dying from persistent CS, but noncardiac organ injury may have also contributed to the decision to withdraw care. These challenges are not unique to this analysis, but underscore the importance of continued efforts to describe and standardize death classification in CS. Finally, our analysis is limited to modes of in-hospital death, which may differ from modes of death among those who survive to hospital discharge.

## Conclusions

Most in-hospital deaths in patients with CS are related to direct cardiovascular causes, particularly persistent CS. However, there is potentially important heterogeneity in modes of in-hospital death across subgroups defined by preceding CA and use of tMCS, which may be relevant to developing new therapeutic approaches, designing randomized trials, and ultimately improving outcomes in CS.

## Figures and Tables

**Table 1:** Modes of Death Definitions

Cardiovascular
Death owing to **heart failure or cardiogenic shock** refers to a death occurring in the context of clinically worsening symptoms and/or signs of heart failure, regardless of heart failure etiology.*
Death owing to **arrhythmia** refers to a death witnessed and attributed to an identified arrhythmia (eg, captured on an electrocardiographic recording, witnessed on a monitor, or unwitnessed but found on implantable cardioverter defibrillator review).^†^
Death owing to **stroke** refers to death after a cerebrovascular event that is either a direct consequence of or substantially contributed to by the stroke.
**Other cardiovascular** death refers to a cardiovascular death not included in the above categories but with a specific, known cause (eg, cardiovascular procedure, cardiovascular hemorrhage, pulmonary embolism, limb ischemia).
Noncardiovascular
Death owing to **anoxic brain injury** refers to death after cardiopulmonary arrest with evidence of severe neurological injury (eg, bilateral absence of pupillary and corneal reflexes ≥96 hours after cardiac arrest, highly malignant EEG pattern, global ischemic pattern on CT scan of the brain or brain MRI), including withdrawal of life-sustaining therapies owing to poor neurological prognosis.
Death owing to **respiratory failure** refers to a death occurring in the context of insupportable oxygenation or ventilation on maximum ventilator settings or inability to liberate from mechanical ventilation, and not primarily owing to heart failure.
Death owing to **noncardiogenic shock** refers to a death occurring in the context of severe hypotension or vasopressor dependence and systemic malperfusion (eg, severe sepsis), and not primarily owing to cardiogenic shock.^‡^
**Other noncardiovascular** death refers to death with a specific cause that is not thought to be cardiovascular in nature and is not respiratory failure, anoxic brain injury, or noncardiogenic shock (eg, renal failure in a patient who is not a candidate for renal replacement therapy).

* Note: Death resulting from mixed shock with a primarily cardiogenic component should be considered death owing to heart failure or cardiogenic shock.

† Note: Terminal agonal rhythms or asystole as part of death from other causes do not meet this definition.

‡ Note: Death resulting from mixed shock with a primarily distributive or hypovolemic component should be considered death owing to noncardiogenic shock.

CT, computed tomography; EEG, electroencephalography; MRI, magnetic resonance imaging.

**Table 2:** Clinical Characteristics of Patients With Cardiogenic Shock Who Died During the Index Hospitalization (*n* = 337)

Clinical Characteristics	No. (%) or Median (Q1-Q3)
Demographics	
Age, years	70 (62–78)
Female sex	116 (34.4%)
Race	
White	174 (66.7%)
Black	59 (22.6%)
Other	28 (10.7%)
Body mass index, kg/m^2^	28 (24–33)
Comorbidities	
Current smoker	53 (15.7%)
Diabetes mellitus	143 (42.4%)
Coronary artery disease	140 (41.5%)
Prior heart failure	145 (43.0%)
Historical LVEF	
≥50%	33 (22.9%)
40 to <50%	13 (9.0%)
<40%	90 (62.1%)
Unknown	9 (6.2%)
Active cancer	30 (8.9%)
Chronic kidney disease	119 (35.3%)
On chronic RRT	31 (26.1%)
Significant pulmonary disease	49 (14.5%)
Significant liver disease	12 (3.6%)
Shock Characteristics	
Etiology	
AMI-CS	120 (35.6%)
HF-CS	170 (50.4%)
Secondary CS*	47 (13.9%)
Presenting LVEF	
≥50%	45 (13.4%)
40% to <50%	23 (6.8%)
<40%	219 (65.0%)
Unknown	50 (14.8%)
Preceding cardiac arrest	111 (32.9%)
Illness severity	
SOFA score	9 (6-12)
SCAI shock classification	
B	18 (5.4%)
C	130 (38.7%)
D	125 (37.2%)
E	63 (18.8%)
Shock management	
Vasoactive medications, maximum number	2 (2–3)
Temporary MCS	132 (39.2%)
IABP	84 (24.9%)
Impella (CP, 5.0, 5.5, RP)	45 (13.4%)
TandemHeart	7 (2.1%)
VA-ECMO	21 (6.2%)
Surgical (nondurable) VAD	3 (0.9%)
Other critical care interventions	
Mechanical ventilation	226 (67.1%)
Acute RRT	78 (23.1%)
Care transitions	
Transitioned to CMO before death	226 (67.1%)

Categorical variables are shown as counts and percentages, and continuous variables as medians with 25th–75th percentile ranges.

* Secondary CS refers to nonmyocardial CS.

AMI, acute myocardial infarction; CS, cardiogenic shock; eGFR, estimated glomerular filtration rate; HF, heart failure; IABP, intra-aortic balloon pump; LVEF, left ventricular ejection fraction; MCS, mechanical circulatory support; RRT, renal replacement therapy; SCAI, Society for Cardiovascular Angiography and Intervention; SOFA, Sequential Organ Failure Assessment; VA-ECMO, venoarterial extracorporeal membrane oxygenation; VAD, ventricular assist device; VIS, vasoactive-inotropic score.
